# The impact of social quarantine on the living status and mental health of the elderly in the Wuhan community: one year after Wuhan COVID-19 blockade

**DOI:** 10.1186/s12877-022-03560-z

**Published:** 2022-11-25

**Authors:** Lisha Dai, Fang Xiong, Wentian Li

**Affiliations:** 1 Psychosomatic Ward, Wuhan Mental Health Center, Wuhan, 410012 Hubei province China; 2 Psychosomatic Ward, Wuhan Hospital for Psychotherapy, Wuhan, 410012 Hubei province China; 3 Tongji Medical College, Huazhong University of Science and Technology, Wuhan, 430074 Hubei province China; 4 China University of Geosciences, Wuhan, 430074 Hubei province China

**Keywords:** COVID-19, Social quarantine, Wuhan community, Older adults, Mental health

## Abstract

**Purpose:**

In order to control the corona virus disease-2019 (COVID-19) pandemic, many countries have adopted social quarantine policies, with older adults in Wuhan suffering the longest and most severe conditions. But few studies have explored the impact of this on the mental health of older adults in Wuhan. The purpose of this paper is to examine changes in the residential status and mental health of this group when 1 year after the social isolation policies in Wuhan.

**Method:**

A cross-sectional study with convenience sampling was conducted to assess the questionnaire of older adults in a total of 21 streets in 5 central and 2 distant urban districts of Wuhan. Using a self-compiled living status questionnaire, the Patient Health Questionnaire-9, the General Anxiety Disorder-7, the PTSD Checklist-Civilian Version, the UCLA Loneliness Scale and the Social Support Rating Scale, our survey evaluated the living status, depression, anxiety, post-traumatic stress symptoms, loneliness and social support of all the participants.

**Results:**

A total of 400 valid samples were obtained. One year after experiencing social isolation, older adults had not changed much from their pre-epidemic living status and mostly lived with their partners. They had satisfactory social support (33.86 ± 6.92) and low levels of depression (3.12 ± 4.30), anxiety (1.52 ± 3.19) and post-traumatic stress symptoms (21.41 ± 7.39), but there were moderate levels of loneliness (38.27 ± 9.31). Among them, depression, anxiety and post-traumatic stress symptoms were significantly higher (ps < 0.05) in older adults who were COVID-19 close contacts while experiencing social isolation.

**Conclusion:**

One year after experiencing Wuhan’s harsh social isolation, older adults in the Wuhan community did not experience significant symptoms of depression, anxiety or post-traumatic stress, but loneliness has increased and the mental health of older adults who were COVID-19 close contacts needs attention.

**Supplementary Information:**

The online version contains supplementary material available at 10.1186/s12877-022-03560-z.

## Background

With more than 400 million confirmed COVID-19 patients and more than 6 million deaths worldwide by April 2022, people around the world are facing the same challenge. COVID-19 not only causes physical damage to humans, but also poses a psychological threat. A review published in The Lancet showed that the global prevalence of depression increased by 27.6% and anxiety increased by 25.6% under the impact of the COVID-19 epidemic [1]. The study further noted that important factors influencing the prevalence of depression and anxiety were the increased prevalence of COVID-19 infections and low mobility of people.

After the outbreak of COVID-19, due to the absence of definitive treatments and vaccines, most countries called for social distancing and a policy of social quarantine to respond to the pandemic situation, especially in China [2]. These measures had a substantial positive impact on the reduced transmission of COVID-19, but had also shown a negative impact on people’s mental health [3, 4]. A multicenter and multinational survey suggested that the number of days spent in quarantine strongly correlated to the perceived stress [4]. This situation was in congruence with Middle East Respiratory Syndrome (MERS) and Severe Acute Respiratory Syndrome (SARS) [5, 6]. Of all the populations affected, the physical and mental health of older adults have attracted the attention of researchers [7]. First, quarantine policies made the lives of elderly who only has limited social activities even worse, and almost impossible to contact outside people. Some researchers claim that social isolation of older adults is a serious public health problem [7]. Second, previous studies have found that in the early 2020 outbreak, the older population had higher fatality rates once being infected [8, 9]. Accordingly, the outbreak of COVID-19 has increased health concerns among older adults. The World Health Organization has also pointed out that the mental health of older people will suffer tremendously and in the long term if no measures are taken. Meng Hui et al. conducted a survey on the mental health status of 1556 older adults in China during the early stages of the outbreak and showed that 37.1% of them felt significant depression and anxiety [10]. In Qiu et al.’s study, emotional problems were more severe in older adults [11]. Based on these current conditions, researchers have proposed a series of strategies to maintain the mental health of the elderly, such as providing telemedicine services and using advertising and publicity to increase the awareness of caring for the elderly [12].

Of all the elderly people in the world, those in Wuhan, have been segregated the longest, the most radical, and their lives have been most affected. On the one hand, to prevent the spread of COVID-19, the Chinese government took a series of measures in Wuhan, Hubei Province, on January 23, 2020, including a city blockade and a ban on going outside. The city blockade coincided with the Chinese New Year, and the policy social isolation may have led to increased loneliness among the elderly. On the other hand, the forbidden to go outside reduces the mobility of the elderly, which may lead to more frailty and more worry about COVID-19, triggering negative emotions such as anxiety and depression. A year has passed since the blockade of Wuhan. The epidemic in China has been recurring, but the overall trend is stable, and life in Wuhan has returned to a relatively normal situation. Existing studies have focused on changes in the mental health of older adults during the epidemic, few have reported how the mental health of the elderly developed, and how the city blockade and social quarantine have affected the residential status of older adults in Wuhan community.

This study aims to use a questionnaire survey to assess the community elderly in Wuhan, to understand the living status and major difficulties faced by this group during the epidemic, to assess the psychological conditions 1 year after the city blockade, including depression, anxiety, post-traumatic stress symptoms, loneliness, and social support, and to provide data to support the maintenance of the psychological health of the elderly and further improve the strategies of protecting psychological health of the elderly.

## Methods

### Participants

From January to March 2021, a total of 21 street from 5 central and 2 distant districts of Wuhan were selected using a convenience sampling to conduct questionnaire assessments of older adults in the community. The inclusion criteria are as follows: 1) 60 years old and above, 2) Lived in Wuhan on January 23, 2020 and experienced the city blockade during the epidemic, 3) Voluntary participation in survey, 4) No conscious impairment, able to communicate normally with investigators, 5) Not COVID-19 confirmed case. Since the confirmed of COVID-19 significantly affects the psychological status of older adults, in order to avoid the effect of this important influencing factor [13], the subjects of this study were only community-dwelling older adults who were not infected with COVID-19. Informed consent was given to the elderly who met the enrollment criteria, and 400 valid questionnaires were finally obtained. This study was approved by the Ethics Committee of Wuhan Mental Health Center affiliated with Tongji Medical College of Huazhong University of Science and Technology.

### Measurement

Among the elderly, it is common that there is some hearing and vision loss, and some people have low level of education and cannot complete the questionnaire. In order to ensure the heterogeneity of the subjects, the investigators conducted a household survey of this group, and for those who could not fill out the questionnaire by themselves, the content of the questionnaire was read aloud in strict accordance with the guideline of the questionnaire to help them to fill out the questionnaire. All questionnaires were in simplified Chinese.

### Living situation assessment

Use 8 self-compiled entries to evaluate information such as living status, caregiving etc. before, during, and after the epidemic. For example, did you encounter difficulties in life during the epidemic? Can anyone help with this? Who did you live with before, during, and after the epidemic? Where did you live? Several options are provided for multiple selection. Based on previous interviews with older adults in the community, five community workers, three psychologists, two physicians and four medical students revised these entries. Further validation was done by 12 older adults who had quarantine experience during the COVID-19 pandemic in Wuhan. The final version of these entries was then formed. It was ensured that the wording was simple and clear, the options were comprehensive.

### Patient Health Questionnaire-9 (PHQ-9)

The PHQ-9 is a depressive symptom assessment scale widely used in clinical and social research and contains 9 items [14]. The Chinese version of PHQ-9 showed good psychometric properties [15]. The response options of each item ranged from 0 (not at all) to 3 (almost every day). The higher total score indicates increased depression: 0–4, 5–9, 10–14, 15–19 and 20–27 is divided into no, mild, moderate and severe depressive symptoms respectively.

### General Anxiety Disorder – 7 (GAD-7)

The GAD-7 is a widely used scale to assess severity of general anxiety and consists of 7 items [16]. The response options of each item ranged from 0 (not at all) to 3 (almost every day). The higher total score indicates increased anxiety: 0–4, 5–9, 10–14 and 15-21v is divided into no, mild, moderate and severe anxiety. The Chinese version of GAD-7 was developed and proved to present reasonable psychometric properties [17].

### PTSD Checklist-Civilian Version (PCL-C)

The PCL-C is a 17-item self-report instrument designed to assess symptoms of posttraumatic stress disorder over the prior 30 days ranging from 1 (not at all) to 5 (extremely), with possible total score ranging from 17 to 85 [18]. The higher the total score, the greater the impact of the traumatic event on the subject. A PCL-C total score of greater than or equal to 50 predicts a possible clinical diagnosis of PTSD, with an efficient balance of sensitivity and specificity (equal to 0.82 and 0.83, respectively )[19]. The psychometric properties of Chinese version of PCL-C are also good [20].

### UCLA Loneliness Scale (UCLA)

The UCLA is a 20-item (11 positive and 9 negative) self-report instrument to assess loneliness in more than student groups [21]. The items are rated on a four-point scale ranging from 1 (never) to 4 (always). A higher score indicates a stronger sense of perceived loneliness: 20–34 points to low loneliness, 35–49 points to moderate loneliness, 50–64 points to upper-middle loneliness and 65–80 points to high loneliness [22]. The Chinese version of the UCLA has been shown to have good reliability and validity [23].

### Social Support Rating Scale (SSRS)

SSRS is a self-report instrument developed by Xiao Shuiyuan in 1993 and contains 10 items [24]. This scale consists of three factors: subjective support, objective support and utilization of support. There are four options ranging from 1 to 4 in each item. Higher scores indicate higher social support and above 30 represent satisfactory social support. This scale is developed base on the background of China and has good psychometric properties in Chinese population.

### Analysis

First, we performed univariate description of the study variables. Using ANOVA to explore the effects of demographic variables and living situations on the mental health status of the elderly in Wuhan community. Finally, we conducted regression analysis to study the important factors that played a major role in depression, anxiety, post-traumatic stress symptoms, loneliness and social support.

## Results

### Demographic statistics

As shown in Table 1, there were 400 elderly people in Wuhan community who participated in the survey and aged 60–96 (71.94 ± 8.40). The sample included 155 male and 245 female. Among the 397 subjects who provided information on their current marital status, 4 were unmarried, 264 were married, 2 were separated, 15 were divorced, and 112 were widowed. 30 of them are close contacts of COVID-19 patients.

**Table 1:** Demographics of Wuhan community elder adults (*n =* 400)

variable	category	N	%
age	60–69	185	46.30
	70–79	113	28.20
	80–89	96	24.00
	90-	6	1.50
gender	Male	155	38.80
	female	245	61.20
education	Did not go to school	49	12.30
	Primary school	83	20.80
	Junior high school	120	30.00
	High school	115	28.70
	Undergraduate and above	33	8.30
marriage	unmarried	4	1.00
	married	264	66.00
	separated	2	0.50
	divorced	15	3.75
	widowed	112	28.00
	Not filled in	3	0.75
COVID-19 close case	Yes	30	7.50
	no	370	92.50
Current living status	Live alone	85	21.63
	Live with their partner	152	38.68
	Live with their children only	51	12.98
	Live with their partner and children	102	25.95
	Live with their parents	3	0.76

### Living situation

#### Pre-pandemic

Of the 376 people who filled out this part of the information, 76 lived alone, 148 lived with their partner, 46 lived with their children only, 100 lived with their partner and their children, 5 lived with their parents, and 1 lived in a nursing home.

#### During pandemic (during the blockade)

Thirty-three people have experienced home isolation, 18 people have experienced hotel isolation, 2 people have lived in square cabins, and 4 people have been hospitalized.

We further investigate the main difficulties these people have encountered during the blockade. The difficulties encountered by the most people were difficulties in purchasing living materials, 207 people encountered such difficulties, 160 people encountered difficulties in purchasing food, 147 people encountered difficulties in purchasing medicines, 87 people encountered difficulties in seeing a doctor, 67 people encountered difficulties in purchasing protective materials, 31 people encountered difficulties in taking care of family members who were sick.

We also explore the social resources that they rely on when encountering these problems. The most important way of seeking help is for children, with 268 people, the second is to solve problems by themselves, with 218 people, 180 people rely on their partners, 121 people seek help from the community, 60 people seek help from relatives, 28 people seek help from their neighbors, 27 people who turned to friends, 7 people who turned to agencies.

#### Current living status

Three hundred ninety-three people filled out this information, of which 85 live alone, 152 live with their partner, 51 live with their children only, 102 live with their partner and children, and 3 live with their parents.

There are 373 people who both provide their living status before and after the epidemic. 9 people had changes in their living status, one of whom changed from living alone to living with at least one relative, and one of them changed from living with at least one relative to living alone, and the rest lived with at least one relative before and after the blockade.

### Descriptive statistics

Means and standard deviations for the study variables are presented in Table 2. This group has satisfactory social support, low level of depression, anxiety and post-traumatic stress symptoms, but there were moderate levels of loneliness. Based on established categories of depression, anxiety, post-traumatic stress symptoms and loneliness, 7.75% of the sample reported moderate or higher depression symptoms, 4.5% reported moderate or higher anxiety symptoms, 1.00% reported possible of a clinical diagnosis of PTSD, 64.75% reported significant or more intense experiences of loneliness.

**Table 2:** The influence of sociodemographic variables on mental health

variable		PHQ-9	GAD-7	PCL-C	UCLA	SSRS
	Mean ± SD	3.12 ± 4.30	1.52 ± 3.19	21.41 ± 7.39	38.27 ± 9.31	33.86 ± 6.92
gender	T	2.208	1.407	2.546	−0.764	−0.674
	p	**0.028**	0.160	**0.011**	0.445	0.501
age	F	1.673	0.249	0.219	2.822	20.313
	p	0.189	0.780	0.803	0.061	**0.000**
education	F	5.899	1.846	2.323	5.605	10.322
	p	**0.000**	0.119	0.056	**0.000**	**0.000**
marriage	F	5.769	2.058	7.864	3.648	50.832
	p	**0.003**	0.129	**0.000**	**0.027**	**0.000**
COVID-19 close case	T	4.574	6.161	7.360	0.531	0.134
	p	**0.033**	**0.013**	**0.007**	0.467	0.715
Current living status	F	5.884	1.838	3.559	2.700	39.648
	p	**0.001**	0.140	**0.014**	**0.045**	**0.000**

Note: *PHQ-9* Patient Health Questionnaire-9, *GAD-7* General Anxiety Disorder – 7, *PCL-C* PTSD Checklist-Civilian Version, *UCLA* UCLA Loneliness Scale, *SSRS* Social Support Rating Scale, *T* T-value for independent samples t-test, *F* F-value for ANOVA, *p* Probability value for t-test or ANOVA and *p* < 0.05 is viewed as statistically significant in this study

### The influence of demographic variables and living status on mental health

In the marital status, 4 people were unmarried and 2 are separated. In the current living status, 3 people live together with their parents, and the number of people was too small to be representative. Therefore, these 3 categories were not included in the following analysis.

As shown in Table 2, gender, education, marital status, current living status, and close contact with COVID-19 patients significantly affected the depression of the elderly (ps < 0.05). Depression levels were significantly higher among women, those who had not attended school, widowed, close contact with COVID-19, and lived alone.

The above demographic factors were used as independent variables for regression analysis, and the results showed that marriage, close contact with COVID-19 and education can significantly predict depression in the elderly (see Supplementary appendix Table S1).

For anxiety, only in close contact with COVID-19 was a significant impact factor (see Table 2).

Gender, marital status, close contacts of COVID-19 and current living status were significant influencing factors of post-traumatic stress symptoms in this group. Post-traumatic stress symptoms were significantly higher among female, widowed, COVID-19 close contact and living alone older adults. The results of regression analysis showed that marriage and COVID-19 close contact significantly predicted post-traumatic stress symptoms (see Supplementary appendix Table S2).

There are significant differences in loneliness among the elderly with different educational levels, marital status and current living status. Among them, the loneliness of the elderly who did not go to school is the highest, and the loneliness of the elderly who live with their partners and children is the lowest. Loneliness is significantly higher among widowed older adults than those who were married. The results of regression analysis showed that education significantly predicted the loneliness (see Supplementary appendix Table S3). Older adults with secondary school education or higher feel less loneliness.

There were significant differences in the social support felt by older adults with different ages, educational levels, marital status, and current residential status. Older adults in the 60–69 age group, with a bachelor degree or above, married, and living with their partner and children feel significantly higher social support. The results of regression analysis showed that age, educational level, and living status significantly predicted social support in older adults (see Supplementary appendix Table S4).

## Discussion

This study assessed the changes in living status and mental health experienced by community-dwelling elderly people who were not infected with COVID-19 in Wuhan and the main difficulties encountered during the social quarantine period of the city blockade. The results showed that the living status of the elderly in Wuhan community did not change much before and after the COVID-19 outbreak, and only 9 people in the sample had changes in living status. In addition, 1 year after the outbreak of the epidemic, the depression, anxiety and post-traumatic stress symptoms of the elderly in Wuhan were low, but the loneliness scale score was 38.27 ± 9.31, which was a moderate degree of loneliness, and this group had satisfactory social support.

In previous studies, in the initial stage of the COVID-19 outbreak in China, the mental health of Chinese residents, especially the elderly, was greatly negatively affected. Studies of the early days of the COVID-19 outbreak reported that 16.5% of the general population experienced moderate to severe depression and 28.8% experienced moderate to severe anxiety [25]. Among the more affected older population, 37.1% experienced depression and anxiety [10]. Before the outbreak of the COVID-19, the incidence of depression among the elderly in China from 2010 to 2019 was 25.55% [26], the incidence of anxiety was 25.2% in 2016 [27], and 11.77% in 2018 [28]. It can be seen that the outbreak of the epidemic has indeed affected the mental health of the elderly. In this study, the levels of depression, anxiety and post-traumatic stress symptoms were not high among the elderly in Wuhan community, only 7.75% of the sample reported moderate or higher depression symptoms, 4.5% reported moderate or higher anxiety symptoms, 1.00% reported possible of a clinical diagnosis of PTSD. This is consistent with previous studies showing that mental health of older adults gradually improves over time [13].

In addition, the results of this study are different from previous studies, the negative emotions of the elderly in the community after the quarantine seem to show a lower level compared to before. On the one hand, as explained by Yifei Yan et al .[13], as the elderly enter their twilight years and are motivated by their limited lives, they are more able to cope with the virus through better emotional regulation [29]. On the other hand, the elderly has rich life experience, and their emotional stability and psychological resilience are better than those of the young [30]. Therefore, in the face of the epidemic, the elderly is a vulnerable group and will also show a higher level of resilience and vitality [31]. The results of this study showed that the elderly in Wuhan community had satisfactory social support, which also indirectly supports this explanation. The SSRS used in this study includes three dimensions: objective support, subjective support and support utilization, and comprehensively evaluates different aspects of social support for the elderly. In previous studies, the total score of the social support rating scale for the elderly was closely related to their mental health status [32]. It is a relatively stable resource to relieve the pressure of the elderly, can increase the positive emotions of the elderly, and provide psychological resources and material assistance [33], which are particularly important during the epidemic. At the beginning of the outbreak, a large number of researchers called for attention to the mental health of the elderly in the community, and made some instructive suggestions, including the establishment of a comprehensive crisis prevention and intervention system that includes epidemiological testing, screening, referral and targeted interventions to reduce psychological distress and prevent further mental health problems [11, 34, 35]. After the outbreak of the epidemic, China took Wuhan as a pilot city for the development of a national social-psychological service system, committed to improving online and offline psychological service platforms, and established municipal and district social-psychological service guidance centers, sub-district (township) social work service centers, and community-based (village) social work service centers. The four-level social-psychological service platform provides mental health services for key and special groups including the elderly. Based on these, a service team composed of mental health professionals, social workers, professional volunteers, etc. has been formed, various mental health lectures and group activities have been organized, and the mental state of the elderly in the community has been assessed and intervened on a regular basis. Negative emotions among the elderly in the community seems to be on a downward trend than before the epidemic, suggesting that the measures taken by Wuhan government may be effective.

It is worth noting that the average level of loneliness among the elderly is moderate 1 year after the blockade, and the proportion of moderate or above loneliness is 64.75%, which is much higher than the 18.8% in previous domestic studies [36], and also higher than 30% in previous studies abroad [37]. This result is not surprising. Although in this study, the elderly still felt the same social support as before the epidemic, and their living status had not changed dramatically, the lifestyles had changed by the epidemic. The elderly in Wuhan is constantly facing social isolation and reduced travel, which limits their social interaction and social participation to a certain extent, and the loneliness of the elderly is increasing day by day. This study showed that education level and living status significantly affect the level of loneliness of the elderly. The elderly who only lived with their children had significantly higher loneliness than those who lived with their partners and children, suggesting that partners have a greater impact on the mental health of the elderly [38].

Regarding the demographic factors that affect the mental health of community elders, consistent with previous researches [10], more attention should be paid to the depression of community elders in females, low education level, widowed and living alone. Female, widowed, and single-living community elders also had relatively higher levels of post-traumatic stress. A multicenter and multinational survey of adults showed no significant differences in PTSD incidence by gender and higher PTSD incidence in widowed individuals [3]. This inconsistency suggests that there are differences in the effects of the quarantine and isolation experience on populations. Future research should explore the effects of segregation policies on different populations and the factors influencing them. In addition, the elderly who were close contact cases had significantly higher level of depression, anxiety or post-traumatic stress than those who were not. It is suggested that this group should still receive attention 1 year after the blockade.

It is important that this study was a cross-sectional study and did not assess the mental health-related data of the elderly in Wuhan before the epidemic, so longitudinal comparisons were not possible. Second, all the questionnaires in this study were self-report scales, which may cause some bias in the results. Future research should combine interviews and qualitative research, and compare with the elderly in the community suffering from the COVID-19, to further explore the development and influencing factors of the mental health of the elderly after the blockade, and to develop a mental health crisis prevention and intervention system more suitable for elderly at this stage to address the challenges of different stages of the epidemic.

Nonetheless, this study is the first to assess the living status and mental health of the elderly in Wuhan community 1 year after the blockade, which has certain implications for future research. First, there are many factors can predict mental health of old adults who have quarantine and isolation experience. These should be considered in future pandemic prevention to better maintain the mental health of older adults. Second, Loneliness is the most prominent negative emotion among these group. More researches and appropriate intervention programs are needed in this area.

## Conclusions

One year after the severe social quarantine in Wuhan in 2019, the living status of the elderly did not change significantly, and the levels of depression, anxiety and post-traumatic stress symptoms were not high, the level of loneliness had increased. Many factors can predict loneliness in this group, including educational level, marital status and current living status. Loneliness was highest among those who had only attended elementary school or had not attended school. The psychological status of the elderly who are close contacted case still needs attention.

## Supplementary Information

**Additional file 1.**

## Data Availability

The datasets used and/or analyzed during the current study are available from the corresponding author on reasonable request.
