# Supplementary material for: The impact of social quarantine on the living status and mental health of the elderly in the Wuhan community: one year after Wuhan COVID-19 blockade
Source: BMC Geriatr. 2022 Nov 25;22:903. doi: 10.1186/s12877-022-03560-z (PMC9700887; doi:10.1186/s12877-022-03560-z)
Supplement: Supplementary file 1 — Additional file 1. [file 12877_2022_3560_MOESM1_ESM.docx]

Supplementary materials

**Table S1 Regression analyses of demographic variables and living status as predictors of depression**

| independent | B | St. Error | β | t | p |
| --- | --- | --- | --- | --- | --- |
| Constant | 4.919 | 1.182 |  | 4.161 | 0.000 |
| gender  (male=ref.) |  |  |  |  |  |
| female | -0.316 | 0.462 | -0.037 | -0.685 | 0.494 |
| education  (did not go to school=ref.) |  |  |  |  |  |
| Primary school | 0.017 | 0.758 | 0.002 | 0.022 | 0.982 |
| Junior high school | -2.049 | 0.734 | -0.224 | -2.789 | **0.006** |
| High school | -1.698 | 0.758 | -0.182 | -2.239 | **0.026** |
| Undergraduate and above | -2.393 | 0.955 | -0.159 | -2.504 | **0.013** |
| marriage  (married=ref.) |  |  |  |  |  |
| divorced | 1.265 | 1.447 | 0.054 | 0.874 | 0.383 |
| widowed | 0.661 | 1.096 | 0.071 | 0.604 | 0.547 |
| COVID-19 close case (No=ref.) |  |  |  |  |  |
| Yes | 2.120 | 0.809 | 0.131 | 2.620 | **0.009** |
| Current living status (live alone=ref.) |  |  |  |  |  |
| Live with their partner | -1.040 | 1.113 | -0.120 | -0.934 | 0.351 |
| Live with their children only | -1.907 | 0.730 | -0.154 | -2.612 | **0.009** |
| Live with their partner and children | -0.696 | 1.133 | -0.073 | -0.614 | 0.540 |

NOTE: In the independent variable, male, did not go to school, married, no COVID-19 close case, live alone were used as the reference group; B = unstandardized regression weight; β = standardized regression weight; t = t-test statistic for the individual predictor variable; p = probability value for t-test and p < 0.05 is viewed as statistically significant in this study.

**Table S2 Regression analyses of demographic variables and living status as predictors of posttraumatic stress symptoms**

NOTE: In the independent variable, female, married, no COVID-19 close case, live alone were used as the reference group; B = unstandardized regression weight; β = standardized regression weight; t = t-test statistic for the individual predictor variable; p = probability value for t-test and p < 0.05 is viewed as statistically significant in this study.

| independent | B | St. Error | β | t | p |
| --- | --- | --- | --- | --- | --- |
| constant | 21.031 | 1.861 |  | 11.299 | 0.000 |
| Gender  (female=ref.) |  |  |  |  |  |
| male | -1.042 | 0.806 | -0.069 | -1.292 | 0.197 |
| Marriage (married=ref.) |  |  |  |  |  |
| divorced | 5.677 | 2.510 | 0.141 | 2.262 | **0.024** |
| widowed | 2.224 | 1.920 | 0.137 | 1.159 | 0.247 |
| COVID-19 close case (No=ref.) |  |  |  |  |  |
| Yes | 4.311 | 1.415 | 0.153 | 3.048 | **0.002** |
| Current living status (live alone=ref.) |  |  |  |  |  |
| Live with their partner | -0.840 | 1.942 | -0.056 | -0.433 | 0.666 |
| Live with their children only | -1.078 | 1.278 | -0.050 | -0.843 | 0.400 |
| Live with their partner and children | 0.055 | 1.979 | 0.003 | 0.028 | 0.978 |

**Table S3 Regression analyses of demographic variables and living status as predictors of loneliness**

NOTE: In the independent variable, did not go to school, live alone were used as the reference group; B = unstandardized regression weight; β = standardized regression weight; t = t-test statistic for the individual predictor variable; p = probability value for t-test and p < 0.05 is viewed as statistically significant in this study.

| independent | B | St. Error | β | t | p |
| --- | --- | --- | --- | --- | --- |
| Constant | 40.056 | 2.651 |  | 15.112 | 0.000 |
| Education (did not go to school=ref.) |  |  |  |  |  |
| Primary school | -1.030 | 1.700 | -0.044 | -0.606 | 0.545 |
| Junior high school | -4.231 | 1.643 | -0.209 | -2.576 | **0.010** |
| High school | -5.416 | 1.685 | -0.262 | -3.214 | **0.001** |
| Undergraduate and above | -6.640 | 2.128 | -0.199 | -3.121 | **0.002** |
| Marriage (married=ref.) |  |  |  |  |  |
| divorced | 4.945 | 3.245 | 0.096 | 1.524 | 0.128 |
| widowed | 2.007 | 2.457 | 0.097 | 0.817 | 0.415 |
| Current living status (living alone=ref.) |  |  |  |  |  |
| Live with their partner | 2.283 | 2.478 | 0.119 | 0.921 | 0.358 |
| Live with their children only | 0.778 | 1.636 | 0.028 | 0.476 | 0.635 |
| Live with their partner and children | 0.380 | 2.532 | 0.018 | 0.150 | 0.881 |

**Table S4 Regression analyses of demographic variables and living status as predictors of social support**

| independent | B | St. Error | β | t | p |
| --- | --- | --- | --- | --- | --- |
| constant | 38.242 | 3.554 |  | 10.760 | 0.000 |
| age | -0.106 | 0.042 | -0.133 | -2.558 | **0.011** |
| Education (did not go to school=ref.) |  |  |  |  |  |
| Primary school | -1.051 | 1.084 | -0.062 | -0.969 | 0.333 |
| Junior high school | -0.543 | 1.068 | -0.037 | -0.508 | 0.611 |
| High school | 1.412 | 1.116 | 0.094 | 1.265 | 0.207 |
| Undergraduate and above | 3.242 | 1.361 | 0.134 | 2.381 | **0.018** |
| Current living status  (living alone=ref.) |  |  |  |  |  |
| Live with their partner | 4.949 | 1.579 | 0.356 | 3.134 | **0.002** |
| Live with their children only | 2.247 | 1.043 | 0.113 | 2.155 | **0.032** |
| Live with their partner and children | 4.775 | 1.617 | 0.310 | 2.952 | **0.003** |
| marriage  (married=ref.) |  |  |  |  |  |
| divorced | -2.378 | 2.070 | -0.064 | -1.149 | 0.251 |
| widowed | -1.643 | 1.576 | -0.110 | -1.043 | 0.298 |

NOTE: In the independent variable, did not go to school, live alone and married were used as the reference group; B = unstandardized regression weight; β = standardized regression weight; t = t-test statistic for the individual predictor variable; p = probability value for t-test and p < 0.05 is viewed as statistically significant in this study.
